# Galectin-3 is involved in inflammation and fibrosis in arteriogenic erectile dysfunction via the TLR4/MyD88/NF-κB pathway

**DOI:** 10.1038/s41420-024-01859-x

**Published:** 2024-02-20

**Authors:** Guanbo Wang, Ruiyu Li, Chen Feng, Kefan Li, Shuai Liu, Qiang Fu

**Affiliations:** 1 Department of Urology, Shandong Provincial Hospital Affiliated to Shandong First Medical University, Jinan, China; 2 Department of Urology, Shandong Provincial Hospital, Shandong University, Jinan, China; 3 Engineering Laboratory of Urinary Organ and Functional Reconstruction of Shandong Province, Shandong Provincial Hospital Affiliated to Shandong First Medical University, Jinan, China

**Keywords:** Mechanisms of disease, Experimental models of disease

## Abstract

Galectin-3 (Gal-3) is a multifunctional protein that has been linked to fibrosis and inflammation in the cardiovascular system. In this study, we examined the impact of Gal-3 on inflammation and fibrosis in patients with arteriogenic erectile dysfunction (A-ED) and the underlying mechanisms involved. To induce arterial injury, we utilized cuffs on the periaqueductal common iliac arteries of Sprague‒Dawley (SD) rats and administered a high-fat diet to co-induce local atherosclerosis. Our results showed that we successfully developed a novel A-ED model that was validated based on histological evidence. In vivo, the vascular lumen of rats subjected to a high-fat diet and cuff placement exhibited significant narrowing, accompanied by the upregulation of Gal-3, Toll-like receptor 4 (TLR4), and myeloid differentiation primary response protein 88 (MyD88) expression in the penile cavernosa. This led to the activation of nuclear factor kappa B 65 (NF-κB-p65), resulting in reduced intracavernosal pressure, endothelial nitric oxide synthase expression, and smooth muscle content, promoting inflammation and fibrosis. However, treatment with Gal-3 inhibitor-modified citrus pectin (MCP) significantly normalized those effects. In vitro, knocking down Gal-3 led to a significant reduction in TLR4, MyD88, and NF-κB-p65 expression in corpus cavernosum smooth muscle cells (CCSMCs), decreasing inflammation levels. In conclusion, inhibiting Gal-3 may improve A-ED by reducing inflammation, endothelial injury, and fibrosis in the penile corpus cavernosum through the TLR4/MyD88/NF-κB pathway. These findings highlight the potential therapeutic target of Gal-3 in A-ED.

## Introduction

Erectile dysfunction (ED) is a prevalent health issue among men [1]. It is predicted that the total number of men affected could increase to approximately 322 million by 2025 [2]. Age, smoking, diabetes, hypertension, dyslipidemia, metabolic syndrome, and obesity are all risk factors for ED [3]. Sixty to eighty percent of EDs are caused by vascular damage, especially atherosclerosis (AS) [4, 5]. AS causes the narrowing of arteries, resulting in insufficient blood supply to the arteries, which ultimately leads to erectile dysfunction. Prior research has demonstrated that atherosclerosis, smooth muscle loss, and increased collagen levels are the primary causes of ED in men with cardiovascular disease (CVD) [6]. Further research has confirmed that atherosclerotic rabbits are more prone to developing ED than nonatherosclerotic rabbits are [7].

There are fewer studies on AS-induced ED models, probably because it is difficult to establish ED models in rats fed only a high-fat diet [8]. Several studies have utilized bilateral internal iliac artery ligation to create simulated arterial injury. However, the stenosis observed in that ED model is not representative of normal physiological AS stenosis; in addition, it does not readily induce endothelial dysfunction [9]. A model of persistent pelvic ischemia has been utilized in various types of research [10–12]. To induce intimal damage, a balloon is used during the procedure. However, this intentional stripping of the intima cannot replicate natural intimal damage. Additionally, executing this procedure can be challenging. As part of our study, we examined a cuff-induced ED model that is based on endothelial injury and a high-fat diet. This cuff-induced model has been previously validated in other studies [13, 14]. In this study, we placed a cuff around the common iliac artery in rats, which is different from the typical placement around the femoral and carotid arteries. This innovative approach results in a local inflammatory response through chronic stimulation. Additionally, the rats were fed a high-fat diet, which, in combination with the cuff placement, accelerated endothelial damage.

Gal-3, a member of the galectin family, is present in endothelial cells, inflammatory cells, and smooth muscle cells. It plays a vital role in cell proliferation, apoptosis, inflammation, and fibrosis and acts as a key regulator of chronic and acute inflammatory states and fibrosis in various tissues [15, 16]. Gal-3 has been shown to act as a ligand for TLR-4 [17] and found to bind to TLR-4 through its carbohydrate recognition domain; this binding leads to an increase in the release of proinflammatory factors from microglia, but this effect is eliminated when TLR4 was knocked down [17]. Studies have also shown that activating Gal-3 promotes the expression of TLR4, MyD88, and NF-κB-p65, which can lead to inflammation [18]. On the other hand, downregulating Gal-3 can inhibit the TLR4/MyD88/NF-κB signaling pathway, leading to a reduction in myocardial fibrosis and inflammation [19].

To clarify the mechanism of action of Gal-3 in A-ED, we used MCP as an inhibitor of Gal-3. MCP is a low-molecular-weight polysaccharide that is easily absorbed into the circulation by the small intestinal epithelium and has anti-inflammatory and antifibrotic properties [20, 21]. The aim of this study was to validate a new A-ED model and explore the role of Gal-3 in A-ED. For the first time, we verified the role of Gal-3/TLR4/MyD88/NF-κB in A-ED and elucidated the impact of Gal-3 on inflammation, fibrosis, and endothelial function in A-ED. These findings may lead to the use of a new therapeutic approach for treating A-ED.

## Results

### The cuff and high-fat diet caused the artery lumen to narrow, leading to ED in rats

**Fig. 1:**
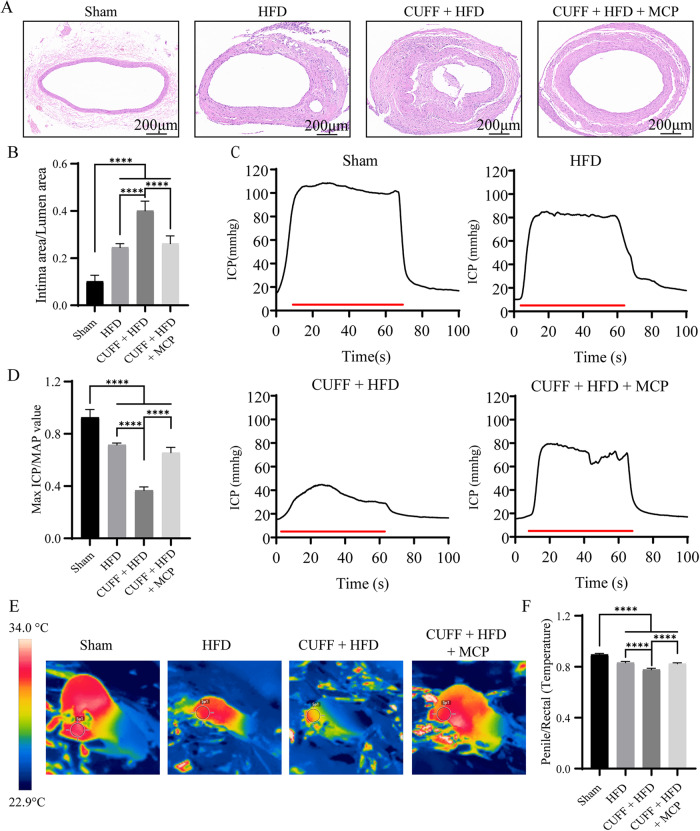
A cuff and high-fat diet caused the artery lumen to narrow, leading to ED in rats. **A** H&E staining of the common iliac artery; scale bar: 200 μm. **B** Ratio of the intima area to the lumen area. **C** ICP curves for each group. The red line indicates 60 s of electrical stimulation of the cavernous nerve. **D** Ratio of the max ICP to the MAP. **E** Real-time observation of penile blood filling during erections in each group of rats. **F** Ratio of penile to rectal temperature. The data are displayed as the mean ± standard deviation (*n* = 6). ****p < 0.0001.

We first established a cuff model in rats and fed the animals a high-fat diet for 12 weeks after surgery. SD rats that received cuffs were divided into two groups and given either MCP or drinking water treatment for 6 weeks. The results showed that, compared with those in the Sham and HFD groups, the lumen in the CUFF + HFD group was significantly narrower, and the corresponding intima-to-lumen area ratio was also significantly higher (Fig. 1A, B). After MCP treatment, the degree of lumen stenosis decreased, and the ratio of the intima area to the lumen area also decreased significantly compared with that in the CUFF + HFD group but was greater than that in the Sham group (Fig. 1A, B). The maximum intracavernous pressure (max ICP) and the max ICP-to-mean arterial pressure (MAP) ratio were significantly lower in the CUFF + HFD group than in the Sham and HFD groups (Fig. 1C, D). However, the max ICP and the ICP-to-MAP ratio in the CUFF + HFD + MCP group were greater than those in the CUFF + HFD group and lower than those in the Sham group (Fig. 1C, D). In addition, compared with rats in the Sham and HFD groups, rats in the CUFF + HFD group had significantly lower blood flow to the penile vessels during an erection, and their penis-to-rectum temperature ratio was lower (Fig. 1E, F). In contrast, compared with the CUFF + HFD group, in the MCP group, there was significant improvement, but the penile-to-rectal temperature ratio was lower in the MCP treatment group than in the Sham group (Fig. 1E, F). These results suggest that A-ED development may be related to vascular remodeling caused by the cuff and high-fat diet. During an erection, vascular remodeling can lead to an inadequate blood supply to the penile vessels. However, these effects can be improved by MCP treatment.

### The altered tissue structure of the corpus cavernosum of the penis

**Fig. 2:**
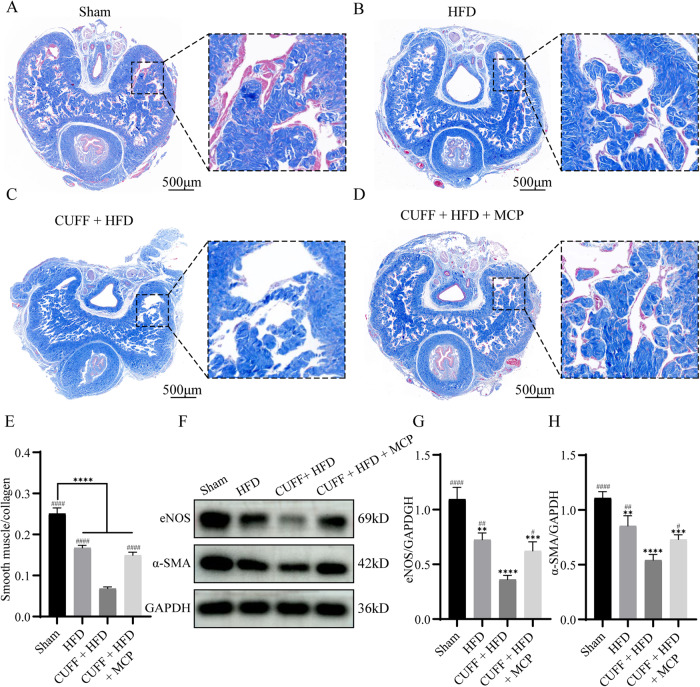
Smooth muscle content and eNOS content in the penile corpus cavernosum. **A**–**D** Masson’s trichrome staining of penile tissue from the various groups. Red indicates smooth muscle, and blue indicates collagen. **E** The bars represent the ratio of smooth muscle to collagen. **F** Representative images of western blots showing the protein expression of eNOS and α-SMA. **G**, **H** The data represent the relative intensities of eNOS and α-SMA. (*n* = 3). **p < 0.01, ***p < 0.001, ****p < 0.0001 compared with the Sham group. # *p* < 0.05, ## *p* < 0.01, #### *p* < 0.0001 compared with the CUFF + HFD group.

We used Masson’s trichrome staining and western blotting to assess histological changes in the penile corpus cavernosum. The penile smooth muscle content and smooth muscle-to-collagen ratio were substantially lower in the CUFF + HFD group than in the Sham and HFD groups (Fig. 2A–E). The penile smooth muscle content and smooth muscle-to-collagen ratio increased significantly after MCP treatment but were lower than those in the Sham group (Fig. 2A–E). In terms of the expression of endothelial nitric oxide synthase (eNOS) and α-SMA, that in the CUFF + HFD group was significantly lower than that in the other groups and increased after MCP treatment (Fig. 2F–H). The results showed that the cuff and high-fat diet caused a decrease in smooth muscle and eNOS levels in rats, damaging the normal physiology of the corpus cavernosum, an effect that was significantly restored after the administration of MCP (Fig. 2F–H).

### Upregulation of Gal-3 in rats with A-ED promoted TLR4/MyD88/NF-κB pathway activation

**Fig. 3:**
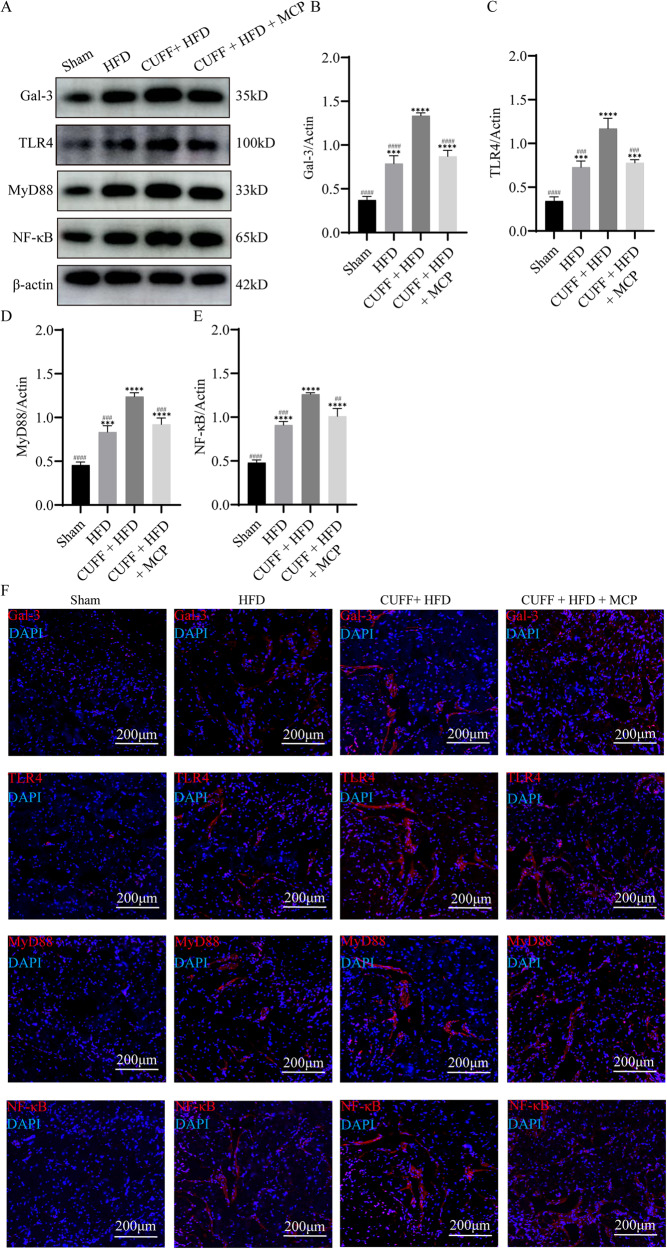
Expression of Gal-3/TLR4/MyD88/NF-κB pathway-related proteins in the penile corpus cavernosum. **A** Western blot showing the expression of the Gal-3, TLR4, MyD88, and NF-κB proteins. **B**–**E** The bars represent the relative intensity of the expression of Gal-3/TLR4/MyD88/NF-κB pathway proteins (*n* = 3). **F** Immunofluorescence data show the expression of the proteins Gal-3, TLR4, MyD88, and NF-κB. Scale bar: 200 μm. ***p < 0.001, ****p < 0.0001 compared with the Sham group. ## *p* < 0.01, ### *p* < 0.001, #### *p* < 0.0001 compared with the CUFF + HFD group.

In addition, to determine whether Gal-3 can regulate the TLR4/MyD88/NF-κB pathway in the rat penis, we used western blotting and immunofluorescence to assess the expression of relevant proteins. Western blot analysis revealed that Gal-3, TLR4, MyD88, and NF-κB-p65 expression was significantly higher in the CUFF + HFD group than in the Sham and HFD groups (Fig. 3A–E). In contrast, the expression of Gal-3, TLR4, MyD88, and NF-κB-p65 was significantly lower after MCP treatment but was greater than that in the Sham group (Fig. 3A–E). Immunofluorescence staining for Gal-3, TLR4, MyD88, and NF-κB-p65 supported these findings (Fig. 3F). These results suggest that Gal-3 expression is elevated in the penile tissue of A-ED rats, further activating the TLR4/MyD88/NF-κB pathway. In contrast, the Gal-3 inhibitor MCP decreased the activation of the TLR4/MyD88/NF-κB pathway by inhibiting Gal-3.

### Upregulation of Gal-3 in the penile tissue of A-ED rats was related to inflammation and fibrosis

**Fig. 4:**
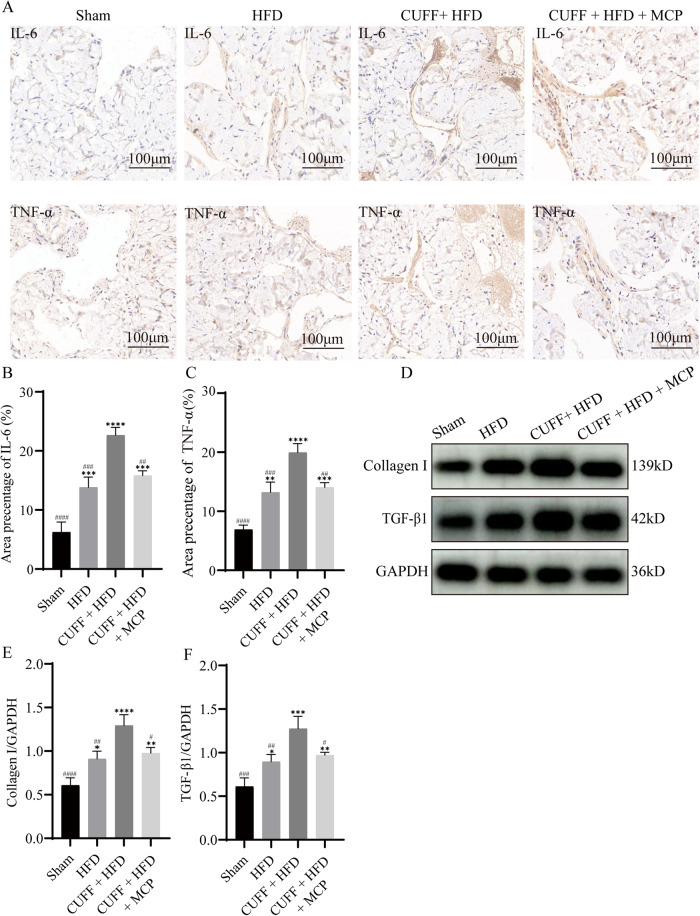
Correlation of the expression of proinflammatory and fibrogenic factors in the penile corpus cavernosum. **A** Immunohistochemical staining in each group for IL-6 and TNF-α. **B**, **C** IL-6- and TNF-α-positive expression area ratio (*n* = 3). **D** Western blot analysis of collagen I and TGF-β1 expression in the rat corpus cavernosum. **E**, **F** Analysis of the relative intensity of collagen I and TGF-β1 expression (*n* = 3). *p < 0.05, **p < 0.01, ***p < 0.001, ****p < 0.0001 compared with the Sham group. # *p* < 0.05, ## *p* < 0.01, ### *p* < 0.001, #### *p* < 0.0001 compared with the CUFF + HFD group.

First, we validated the expression of Gal-3, TLR4, MyD88, and NF-κB-p65, which are important proteins involved in inflammatory fibrosis. Second, we used immunohistochemistry and western blot methods to further examine the expression of relevant inflammatory and fibrosis-related proteins. Immunohistochemical results showed that the expression of the proinflammatory factors interleukin 6 (IL-6) and tumor necrosis factor-alpha (TNF-α) was significantly higher in the CUFF + HFD group than in the Sham and HFD groups (Fig. 4A–C). However, these effects were reversed by MCP treatment (Fig. 4A–C). In addition, western blot analysis confirmed that the expression of transforming growth factor beta1 (TGF-β1) and collagen I (collagen I) was highest in the CUFF + HFD group (Fig. 4D–F). After MCP treatment, the expression of TGF-β1 and collagen I decreased significantly but was still higher than that in the Sham group (Fig. 4D–F).

### Knockdown (KD) of Gal-3 downregulated TLR4/MyD88/NF-κB pathway expression and reduced proinflammatory cytokine levels in CCSMCs

**Fig. 5:**
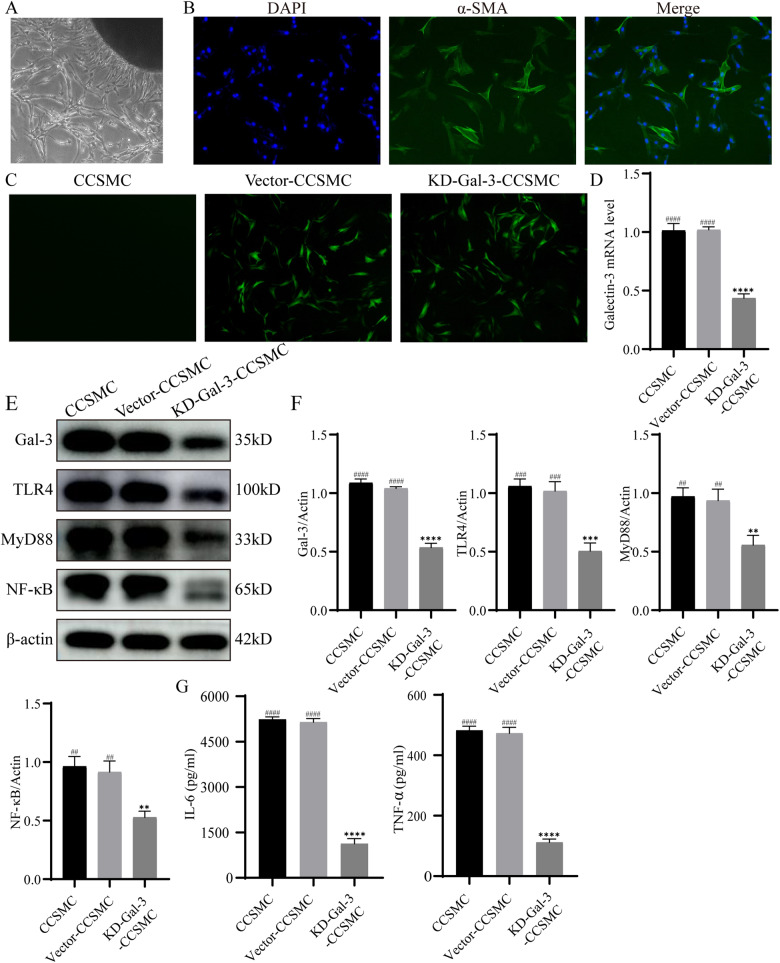
Effect of Gal-3 knockdown on the expression of TLR4/MyD88/NF-κB pathway-related proteins and the levels of proinflammatory cytokines in CCSMCs. **A** After one week of culture, CCSMCs appeared around the cavernous tissue mass. **B** Immunofluorescence identification of CCSMCs (α-SMA). **C** CCSMCs were transfected with lentiviral vectors containing Galectin-3 knockdown lentivirus or the corresponding negative control. **D** qRT‒PCR analysis of Gal-3 in CCSMCs (*n* = 3). **E** Expression of the Gal-3, TLR4, MyD88, and NF-κB proteins in each group of CCSMCs. **F** Relative intensity of the expression of the Gal-3, TLR4, MyD88, and NF-κB proteins (*n* = 3). **G** Levels of IL-6 and TNF-α in the supernatant of each group of CCSMCs (*n* = 3). **p < 0.01, ***p < 0.001, ****p < 0.0001 compared with the CCSMC group. ## *p* < 0.01, ### *p* < 0.001, #### *p* < 0.0001 compared with the KD-Gal-3-CCMSC group.

To further investigate the potential role of Gal-3, we extracted primary CCSMCs and identified them by immunofluorescence (Fig. 5A, B). Third-passage CCSMCs were transfected with a Gal-3 knockdown lentivirus, and RNA was extracted for PCR analysis of Gal-3 (Fig. 5C, D). After the expression of the constructed knockdown CCSMCs was stable, we used Western blotting to assess the expression levels of Gal-3, TLR4, MyD88, and NF-κB. In addition, we used ELISA kits to determine the expression levels of the proinflammatory cytokines IL-6 and TNF-α in the supernatant of CCSMCs. The results showed that the expression of Gal-3, TLR4, MyD88, and NF-κB-p65 was significantly lower in the KD-Gal-3-CCSMC group than in the CCSMC and Vector-CCSMC groups (Fig. 5E, F). In addition, the expression levels of the inflammatory factors IL-6 and TNF-α were significantly reduced in the KD-Gal-3-CCSMC group (Fig. 5G). These in vitro results suggest that the Gal-3/TLR4/MyD88/NF-κB pathway is present in CCMSCs and regulates the levels of inflammatory factors.

## Discussion

ED is a common problem that can have negative effects on the confidence and quality of life of men [22], and there are several etiological factors, including vascular, neurogenic, hormonal, cavernosal, iatrogenic, and psychogenic factors [23]. Of these, vascular causes play important roles in ED [24]. In turn, vascular causes involve atherosclerosis, endothelial dysfunction, and inflammation [25]. This is a relatively complex and worthwhile area to explore. In this study, we established a novel and relatively simple vascular injury model and further confirmed that this model caused A-ED by measuring erectile function via Masson’s trichrome staining (Fig. S1). In this study, we found for the first time that Gal-3, through the TLR4/MyD88/NF-κB pathway, promotes inflammatory responses in penile tissue, cavernous fibrosis, and penile endothelial dysfunction, ultimately leading to decreased erectile function.

In this study, an A-ED model was created by wrapping cuffs around the bilateral common iliac arteries of rats and feeding them a high-fat diet for 12 weeks. After 12 weeks, half of the rats were given MCP for 6 weeks (*n* = 6), and the other half were left untreated (*n* = 6). The results confirmed the reliability of the model, as the lumen of the iliac arteries in the CUFF + HFD rats was significantly narrower than that in the normal rats. Additionally, the corresponding ratios of the maximal ICP to the MAP and the penile-to-rectal temperature ratio were significantly reduced, which is consistent with other A-ED models [26]. A-ED is caused by structural and functional changes, particularly focal lesions that narrow the lumen and cause less blood flow into the artery. This leads to fibrosis of the rat cavernous tissue and may be related to TGF-β1 [6, 9, 27–29]. The normal erectile function of the corpus cavernosum relies on intact endothelial relaxation, which is mediated by nitric oxide (NO) production via eNOS. The dysregulation of eNOS can lead to endothelial dysfunction [30]. Our model validates the observed structural and functional changes in A-EDs. Compared with that in normal rats and rats fed a high-fat diet alone, in the penile corpus cavernosum of CUFF + HFD rats, we found significantly higher expression of TGF-β1 and collagen I, which are associated with fibrosis, and the expression of eNOS and alpha-smooth muscle actin (α-SMA) was lower. Fibrosis was also assessed by Masson’s trichrome staining; there was a significant reduction in smooth muscle content in CUFF + HFD rats, corresponding to a lower smooth muscle-to-collagen ratio than that in normal and high-fat diet rats alone. However, after treatment with MCP, these phenomena improved significantly compared to those in CUFF + HFD rats.

Galectin-3 is involved in regulating important cellular functions and has been linked to various human disorders, including cancer, fibrosis, and chronic inflammation [31]. Elevated levels of Gal-3 have been observed in individuals with fibrosis in multiple tissues, such as the liver, kidney, lungs, and heart [32–35]. In lung fibrosis induced by TGF-β, Gal-3 plays a crucial role [34]. Moreover, in liver fibrosis, the deletion of Gal-3 blocks myofibroblast activation by inhibiting TGF-β [36]. Furthermore, Gal-3 overexpression in rat vascular smooth muscle cells leads to an increase in collagen I expression, and the suppression of Gal-3 by MCP or small interfering RNA prevents collagen I synthesis [37]. Gal-3 has been found to play a role in inflammation. A previous study showed less severe inflammation and lower interleukin-1beta (IL-1β) production in mice lacking Gal-3 than in wild-type mice [38]. Compared with wild-type microglia, in microglia deficient in Gal-3, there was lower production of the cytokines IL-1β and interleukin 12 (IL-12), an effect that may be related to the involvement of Gal-3 in proinflammatory activation via specific inflammatory pathways [39]. Notably, Gal-3 has also been suggested to be a marker of coronavirus disease 2019 (COVID-19) severity, and the inhibition of Gal-3 may reduce inflammation and fibrosis in patients with COVID-19 [40, 41]. Importantly, patients with COVID-19 are at a higher risk of developing ED [42]. It is worth investigating whether Gal-3 leads to ED in COVID-19 patients by causing vascular injury. In this study, we initially validated that Gal-3 causes A-ED through the TLR4/MyD88/NF-κB pathway. We observed higher expression of Gal-3, TLR4, MyD88, and NF-κB-p65 in the penile corpus cavernosum in CUFF + HFD rats than in rats fed normal and high-fat-only diets; the levels of the corresponding proinflammatory cytokines IL-6 and TNF-α and fibrosis markers TGF-β1 and collagen I were also significantly higher in CUFF + HFD rats. However, after 6 weeks of MCP administration, in CUFF + HFD rats, there was a significant reduction in the expression of Gal-3, TLR4, MyD88, and NF-κB-p65 and a decrease in the corresponding expression of TGF-β1, collagen I, IL-6, and TNF-α. In our in vitro experiments, we found that the knockdown of Gal-3 via lentiviral transduction in CCSMSCs resulted in similar decreases in the expression of Gal-3, TLR4, MyD88, and NF-κB-p65 and in the levels of proinflammatory cytokines. These findings suggest that Gal-3 plays an important role in A-ED.

**Fig. 6:**
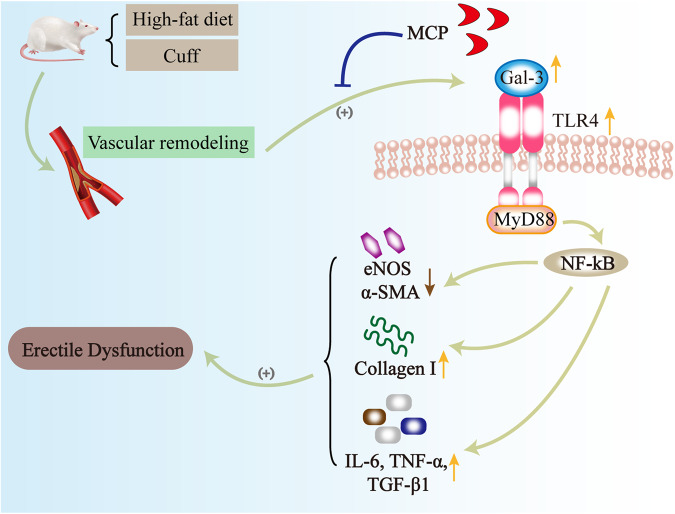
Schematic representation of the mechanism of action of Gal-3 in the A-ED through the TLR4/MyD88/NF-κB pathway. A cuff and high-fat diet caused vascular remodeling and decreased penile blood flow, which in turn upregulated Gal-3 expression and activated the TLR4/MyD88/NF-κB signaling pathway. These changes further caused endothelial dysfunction, inflammation, and fibrosis. This eventually led to A-ED. The Gal-3 inhibitor MCP reversed these phenomena.

Our study identified a new model of arterial erectile dysfunction induced by a high-fat diet and cuff tubes. However, further preclinical research is required to fully understand this model. Additionally, we confirmed the involvement of Gal-3 in A-ED, causing endothelial dysfunction, inflammation, and fibrosis in the penile corpus cavernosum through the TLR4/MyD88/NF-κB pathway (Fig. 6). Our findings suggest that Gal-3 could be a potential therapeutic target for A-ED in the future.

## Materials and methods

### Animal studies

**Fig. 7:**
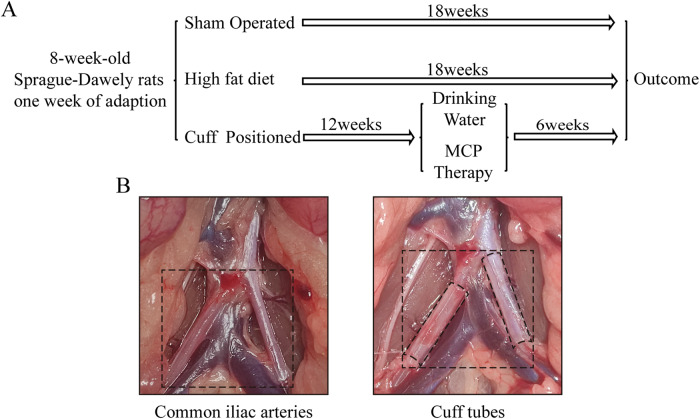
The study protocol and location of the common iliac artery cuff. **A** Time course of animal modeling, diet, and treatment; **B** Local anatomical views of the abdomen of an SD rat; from left to right, the right and left common iliac arteries were dissected, and a cuff was placed around each artery.

Twenty-four 8-week-old male Sprague‒Dawley rats were acquired from Jinan Pengyue Laboratory Animal Breeding Co., Ltd. All animal studies, including animal treatment and handling, were approved by the Institutional Animal Care and Use Committee of Shandong Provincial Hospital (Ethics No. 2022-076). The rats were randomly allocated to cages, housed at 22–24 °C with a 12-h light/dark cycle, and had access to food and water. After one week of acclimatization, the rats were randomly divided into four groups (*n* = 6 per group): Sham operation control group (exposure of the common iliac artery), HFD group (high-fat diet after exposure of the common iliac artery), CUFF + HFD group (high-fat diet after cuff placement around the common iliac artery), and CUFF + HFD + MCP group (cuff placement around the common iliac artery and a high-fat diet for 12 weeks, followed by MCP treatment). Mice in the CUFF + HFD + MCP group were given MCP (100 mg/kg/day) for 6 weeks (Fig. 7A). MCP was dissolved in drinking water because of its good water solubility. The dose of MCP was determined daily according to the amount of water consumed by the rats, and the Sham group was fed a regular diet as a control group. The sample size was estimated based on the results of in vitro pre-tests on animals and previous experience. Rats were included in the study if intraoperative cuff placement was successful and there were no deaths, otherwise, they were excluded. The animals were grouped using the random number table method. Different experimenters were in charge of rat modeling experiments, experimental grouping, and evaluation of experimental results.

### Iliac artery cuff placement

SD rats were anesthetized with Avastin (280 mg/kg). First, the abdomen was cut open, the surrounding fatty tissue was removed, and the two common iliac arteries were separated. The cuff installation method was the same as that used previously [43]. Two polyethylene (PE-160) tubes (5 mm long, 1.14 mm inner diameter, 1.57 mm outer diameter; Becton Dickinson, Franklin Lakes, NJ) were placed loosely around each artery. The muscle and skin were sutured, and the rat was placed back into the cage (Fig. 7B). In the Sham and HFD groups, the two common iliac arteries were separated without cuff placement.

### Infrared ray thermography (IRT)

As described in earlier studies [44], before being placed on the surgery table, the rats were anesthetized. The side of the rat was attached to a FLIR T540 thermal imager (FLIR Systems, Boston, MA), and the penis was positioned in the center of the lens frame. Additionally, a thermometer was gradually placed into the rectum. The operator injected apomorphine (APO, 100 μg/kg) subcutaneously to stimulate an erection. Changes in the corresponding penile temperature during erection of the penis were observed, and the rectal temperature was recorded.

### Measurement of erectile function

After six weeks of therapy, rats were anesthetized with 280 mg/kg of avertin. MAP and maximum ICP were measured. The left carotid artery was then punctured to place a PE-50 tube (Becton Dickinson & Co., Sparks, MD) to measure systemic arterial blood pressure. A 23-gauge needle filled with 250 U/mL heparinized saline solution was used to assess the ICP. After the isolation and identification of the pelvic ganglion, the nerve was stimulated with bipolar electrodes for 60 s at 5 V, 25 Hz, and a 5 ms pulse width. In addition, a BL-420F pressure transducer (AD Instruments, Sydney, Australia) simultaneously recorded variations in MAP and ICP.

### Hematoxylin and eosin (H&E) staining

The iliac vessel was freed from the cuff and preserved in 4% paraformaldehyde for 24 h. The specimens were embedded in paraffin and sectioned (5 µm), and the sections were stained with a hematoxylin-eosin staining kit (Servicebio, China). Image-Pro Plus 5.0 software (Media Cybernetics, Inc., Bethesda, MD, USA) was used to delineate the intima and lumen regions.

### Masson’s trichrome staining

In brief, a Masson’s trichrome staining kit (Servicebio, China) was used to evaluate the expression of collagen fibrils and smooth muscle in the corpus cavernosum tissue (collagenous fibers are colored blue, and smooth muscle is colored red). An image analysis system was used to calculate the proportion of collagen to smooth muscle.

### Real-time quantitative reverse transcription‒polymerase chain reaction (qRT‒PCR)

Total RNA was extracted from CCSMCs using TRIzol reagent (Invitrogen). Reverse transcription was performed using the PrimeScript RT Kit (TaKaRa, Japan). A SYBR Green PCR kit (Toyobo, Osaka, Japan) was subsequently used for qPCR. The total volume of the reaction mixture was 20 µl, which included 10 µl of qPCR MIX, 0.8 µl of each of the forward and reverse primers, 2 µl of cDNA template, and deionized water. The reaction conditions were as follows: initial denaturation at 95 °C for 60 s; 40 cycles of denaturation at 95 °C for 15 s and annealing and stretching at 60 °C for 60 s. The experimental data were analyzed using the 2^–ΔΔCT^ method. GAPDH was used as an internal control. The sequences of primers used for RT‒qPCR are shown in Table 1.

**Table 1:** Primer sequences of qRT-PCR.

Gene	Primer sequences (5’ to 3’)	
Galtein-3	Forward	GCAACACGAAGCAGGACAATAACT
	Reverse	TCATTGACCGCAACCTTGAAGTG
GAPDH	Forward	GGCACAGTCAAGGCTGAGAATG
	Reverse	ATGGTGGTGAAGACGCCAGTA

### Western blot analysis

Briefly, RIPA buffer (Cell Signaling Technology, USA) containing a protease inhibitor cocktail (Cell Signaling Technology, USA) was used to lyse the obtained rat penile tissues or CCSMCs for 30 minutes before centrifugation at high speed for 15 min. The supernatant fraction was aspirated and transferred to another centrifuge tube. A BCA concentration assay kit (Beyotime, China) was used to measure the protein concentration. Identical amounts of protein from different tissues or cell lysates were separated using sodium dodecyl sulfate (SDS)-polyacrylamide gel electrophoresis (PAGE) and then transferred to polyvinylidene fluoride (PVDF) membranes. After overlaying the membranes with 5% fat-free milk, they were incubated overnight at 4 °C with primary antibodies against Galectin-3 (ab227249, Abcam, 1:5000), TLR4 (#66350-1-lg, Proteintech, 1:4000), Myd88 (ab219413, Abcam, 1:1000), NF-κB-65 (#8242, Cell Signaling Technology, 1:1000), GAPDH (#10494-1-AP, Proteintech, 1:10000), β-actin (#20536-1-AP, Proteintech, 1:2500), collagen I (ab270993, Abcam, 1:1000), TGF-β1 (ab215715, Abcam, 1:1000), eNOS (#27120-1-AP, Proteintech, 1:500) and α-SMA (#14395-1-AP, Proteintech, 1:5000). After three washes, the corresponding secondary antibodies were added, and the membrane was incubated for 1 h at room temperature. Enhanced chemiluminescence (ECL) reagents (KeyGEN BioTECH, China) were used to detect protein bands, which were then visualized using an imaging system (UVP GDS-8000, USA).

### Enzyme-linked immunosorbent assay (ELISA)

The supernatant of each group of CCSMCs was collected and centrifuged to removed debris. IL-6 and TNF-α levels were measured using ELISA kits (ABclonal, PK00020; PK00029; China).

### Immunofluorescence

The entire middle part of the penis was fixed by immersion in 4% paraformaldehyde and dehydrated overnight in PBS containing 30% sucrose. The tissue was evenly sliced into 5-µm sections and placed on slides. The sections were processed with primary antibodies against Gal-3 (1:500; Abcam), TLR4 (1:100; Abcam), MyD88 (1:100; Proteintech), and NF-κB-65 (1:500; Cell Signaling Technology) for 1 h. The sections were then washed and treated with Alexa Fluor 594-labeled secondary antibodies (Invitrogen, Carlsbad, CA, USA). To stain the nuclei, the sections were incubated with DAPI (Thermo Fisher, MA, USA) for 5 min. A microscope was then used to analyze the fluorescence.

### Immunohistochemistry

Briefly, embedded penile tissue was sliced into 5-µm sections before being deparaffinized and hydrated with xylene and a gradient ethanol series. Hydrogen peroxide (0.3%; Servicebio, China) was used to quench endogenous peroxidase activity, after which antigen recovery solution was heated to 37 °C to recover the antigen. To block nonspecific binding, 5% goat serum (Servicebio, China) was utilized. The sections were incubated with primary antibodies against TNF-α (1:200; Servicebio) and IL-6 (1:1000; Abcam) overnight at 4 °C. The sections were then incubated with secondary antibodies at room temperature for 1 h before being treated with 3,3’-diaminobenzidine tetrahydrochloride (DAB) for 5 min. For nuclear staining, the sections were counterstained with a hematoxylin solution.

### CCSMC isolation and characterization

CCSMCs were isolated from the spongiosa of normal SD rats (8 weeks) following the methods described in a previous study [45]. Then, a specific volume of Dulbecco’s modified Eagle medium (DMEM) was added to a culture flask (25 cm^2^) containing CCSMCs, which were then incubated in a carbon dioxide (CO_2_) incubator at 37 °C with 5% CO_2_. The cells were passaged at a ratio of 1:3 after they reached a confluence of 80 to 90%. After the third passage, α-SMA (1:1600; Proteintech) immunofluorescence was used to identify the cells.

### Cell transfection

CCSMCs (1 × 10^4^/well) were seeded in 6-well plates. Lentiviral vectors containing Galectin-3 knockdown lentivirus or the corresponding negative controls were purchased from GKN Genetics (Shanghai, China). After incubating the cells with a solution containing lentivirus for 6 h, the supernatant was removed, and fresh culture medium was added. The cells were further incubated in an incubator for three to five days.

### Statistical analysis

The data in this study conformed to the normal distribution and were represented as the mean standard deviation and were gathered from at least three separate studies. With the use of the GraphPad Prism 8.0 software, statistical analysis was carried out using a one-way analysis of variance and post-hoc Tukey’s test for multiple groups or the Student’s *t*-test for two groups. The homogeneity of variance was tested using the Brown-Forsythe test. Differences were deemed statistically significant for *P* < 0.05.

### Supplementary information

Alterations in the structure and function of penile corpus cavernosum tissue.

Alterations in the structure and function of penile corpus cavernosum tissue.

## Data Availability

All the data for this study are presented in the text or supplementary materials.
