# Supplementary material for: Galectin-3 is involved in inflammation and fibrosis in arteriogenic erectile dysfunction via the TLR4/MyD88/NF-κB pathway
Source: Cell Death Discov. 2024 Feb 20;10:92. doi: 10.1038/s41420-024-01859-x (PMC10879531; doi:10.1038/s41420-024-01859-x)
Supplement: Supplementary file 1 — Alterations in the structure and function of penile corpus cavernosum tissue. [file 41420_2024_1859_MOESM1_ESM.docx]

**Figure S1** Alterations in the structure and function of penile corpus cavernosum tissue.

**
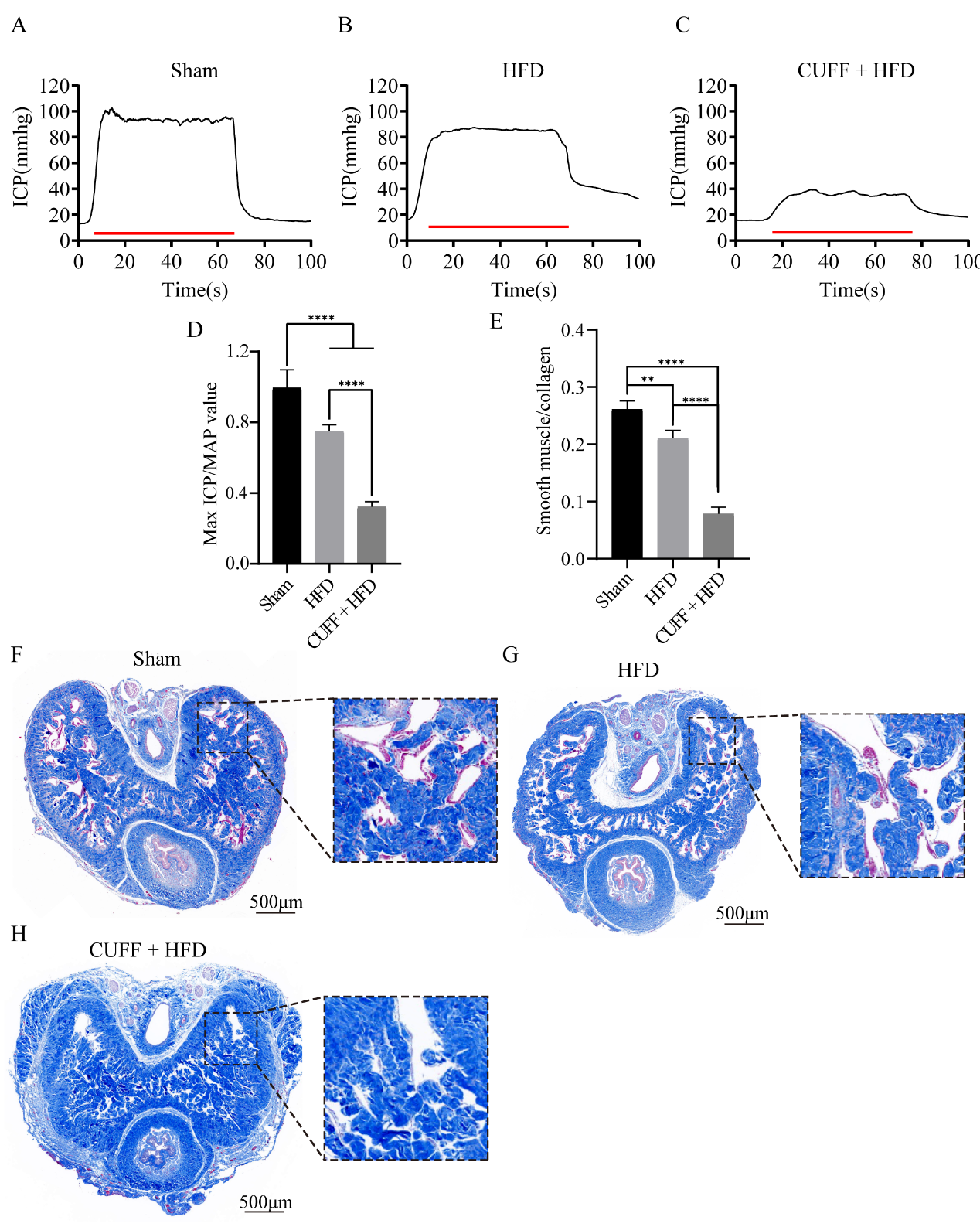
**

(A-C) ICP curves for each group after 12 weeks of modeling. The red line indicates 60 seconds of electrical stimulation of the cavernous nerve. (D) Ratios of Max ICP to MAP (n=6). (E) The bars represent the ratio of smooth muscle to collagen (n=3). (F-H) Masson's trichrome staining of various groups of penile tissue. Red is smooth muscle and blue is collagen. ** p < 0.01, **** p < 0.0001,.
