# Supplementary figures and images for: Galectin-3 is involved in inflammation and fibrosis in arteriogenic erectile dysfunction via the TLR4/MyD88/NF-κB pathway
Source: Cell Death Discov. 2024 Feb 20;10:92. doi: 10.1038/s41420-024-01859-x (PMC10879531; doi:10.1038/s41420-024-01859-x)

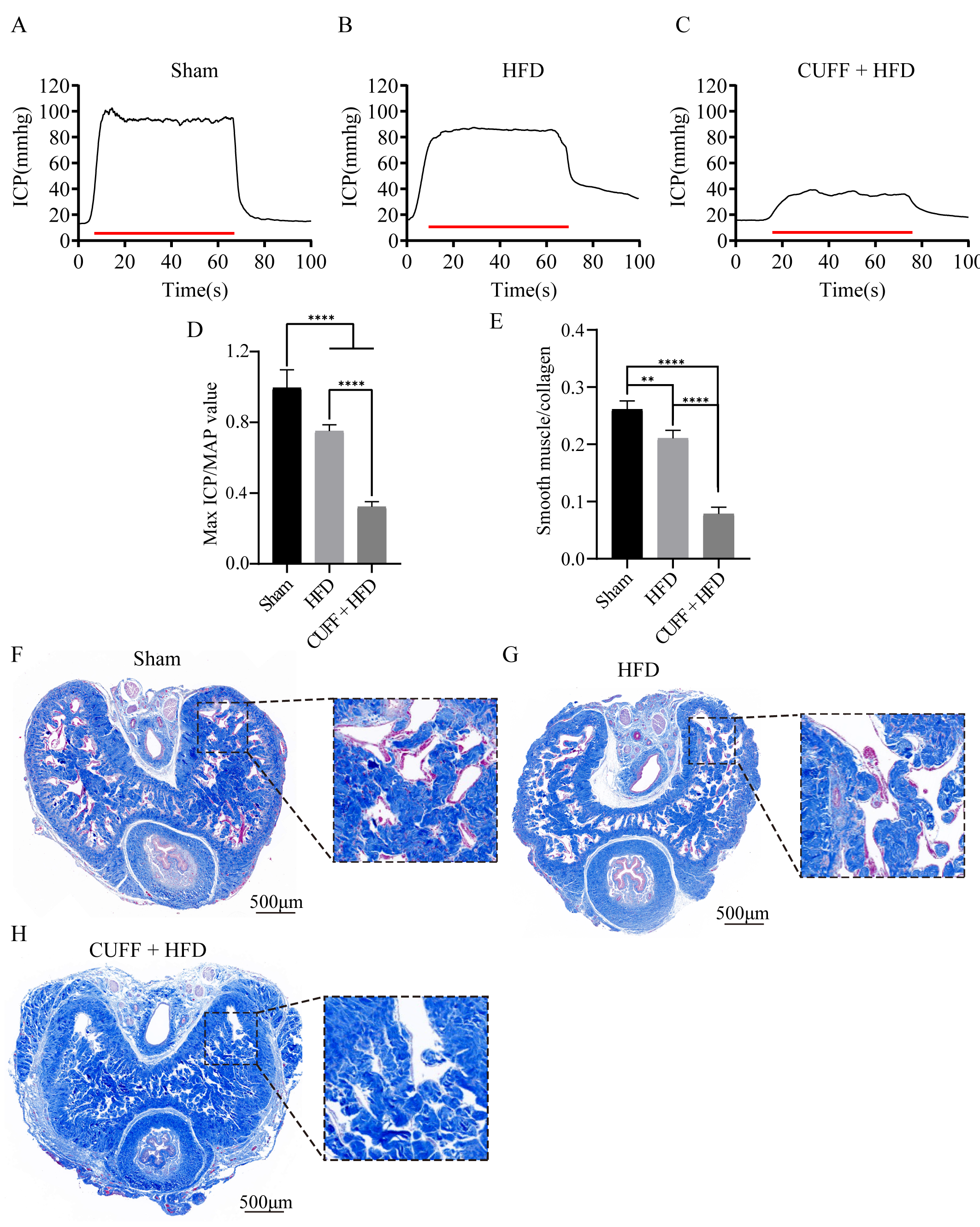

Supplement: Supplementary file 2 — Alterations in the structure and function of penile corpus cavernosum tissue. [file 41420_2024_1859_MOESM2_ESM.tif]
